# Infant Serum and Maternal Milk Vitamin B-12 Are Positively Correlated in Kenyan Infant-Mother Dyads at 1–6 Months Postpartum, Irrespective of Infant Feeding Practice^1–3^

**DOI:** 10.1093/jn/nxx009

**Published:** 2018-01-25

**Authors:** Anne M Williams, Christine P Stewart, Setareh Shahab-Ferdows, Daniela Hampel, Marion Kiprotich, Beryl Achando, Audrie Lin, Clair A Null, Lindsay H Allen, Caroline J Chantry

**Affiliations:** 1 Hubert Department of Global Health, Emory University, Atlanta, GA; 2 Program in International and Community Nutrition, Department of Nutrition, University of California, Davis, CA; 3 USDA, ARS Western Human Nutrition Research Center, Davis, CA; 4 Innovations for Poverty Action, New Haven, CT, USA and Kisumu, Kenya; 5 School of Public Health, University of California Berkeley, Berkeley, CA; 6 Mathematica Policy Research, Washington DC; 7 University of California Davis Medical Center, Sacramento, CA

**Keywords:** vitamin B-12, human milk, micronutrient deficiency, infant feeding, breastfeeding, lactation, Kenya

## Abstract

**Background:**

Vitamin B-12 is an essential nutrient required for many functions including DNA synthesis, erythropoiesis, and brain development. If maternal milk vitamin B-12 concentrations are low, infants may face elevated risks of deficiency when exclusively breastfed.

**Objective:**

We evaluated cross-sectional associations between infant serum vitamin B-12 concentrations and maternal milk vitamin B-12 concentrations at 1–6 mo postpartum among an unsupplemented population in rural western Kenya, and assessed biological demographic, and dietary characteristics associated with adequate infant serum vitamin B-12.

**Methods:**

We modeled *1*) infant serum vitamin B-12 using maternal milk vitamin B-12 concentration with linear regression; and *2*) adequate (>220 pmol/L) infant serum vitamin B-12 using hypothesized biological, demographic, and dietary predictors with logistic regression. In both models, we used generalized estimating equations to account for correlated observations at the cluster-level.

**Results:**

The median (quartile 1, quartile 3) infant serum vitamin B-12 concentration was 276 pmol/L (193, 399 pmol/L) and approximately one-third of infants had serum vitamin B-12 ≤220 pmol/L, indicating that they were vitamin B-12 depleted or deficient. There was a positive correlation between maternal milk and infant serum vitamin B-12 (*r* = 0.36, *P* < 0.001) and in multivariable analyses, maternal milk vitamin B-12 concentration was significantly associated with infant serum vitamin B-12 adequacy (*P*-trend = 0.03).

**Conclusions:**

Despite a high prevalence (90%) of maternal milk vitamin B-12 concentrations below the level used to establish the Adequate Intake (<310 pmol/L), there was a low prevalence of infant vitamin B-12 deficiency. We found few factors that were associated with infant vitamin B-12 adequacy in this population, including infant feeding practices, although maternal vitamin B-12 status was not measured. The contribution of maternal milk to infant vitamin B-12 status remains important to quantify across populations, given that maternal milk vitamin B-12 concentration is modifiable with supplementation. This trial was registered at clinicaltrials.gov as NCT01704105.

## Introduction

Vitamin B-12 is an essential nutrient required for DNA synthesis, erythropoiesis, neurologic functioning, and brain development among other functions (1–3). Exclusively breastfed infants are particularly vulnerable to vitamin B-12 deficiency during lactation if their mothers are not consuming adequate animal source foods, foods fortified with vitamin B-12, or vitamin B-12 supplements (4–6). Infants are especially at risk if maternal status and accumulation of fetal stores were low during pregnancy (7, 8). It has been estimated that fetal demand for vitamin B-12 is 0.3 μg/d (9). Women in resource-poor settings often have low consumption of animal-source foods (10–13) and have been found to have relatively low concentrations of vitamin B-12 in their milk (7, 11, 13–16). In affluent settings, vitamin B-12 status is often lower in breastfed children than in formula-fed children (17–19), most likely due to the high levels of vitamin B-12 in infant formula and cow milk. Exclusive breastfeeding is recommended by the WHO for infants ≤6 mo old (20). Because the vitamin B-12 concentration in human milk fluctuates with maternal intake and status (15), infants are at risk of vitamin B-12 deficiency if milk concentrations are low and stores from birth are suboptimal (21, 22).

The Institute of Medicine defines the Adequate Intake (AI) of vitamin B-12 for infants 0–6 mo to be 4 μg/d, although this estimate is based on a small sample of nonsupplemented Brazilian women with apparently healthy infants (2). The concentration of human milk vitamin B-12 necessary for exclusively breastfed infants to attain the daily AI is estimated to be 310 pmol/L (2, 13).

We previously reported a median milk vitamin B-12 concentration of 113 pmol/L among Kenyan women between 1 and 6 mo postpartum (13). Given that the mothers’ milk vitamin B-12 concentration was low, we sought to assess serum vitamin B-12 concentrations in their infants and evaluate the association with mother's milk vitamin B-12 among this unsupplemented population in rural western Kenya. We hypothesized that maternal milk and infant serum vitamin B-12 concentrations would be positively correlated. A secondary objective of this research was to explore associations between biological, demographic, and dietary characteristics hypothesized to be associated with infant vitamin B-12 adequacy.

## Methods

### 

#### Study participants and ethical considerations

Milk and serum samples were collected from mothers and infants participating in a biochemical substudy of the Water Sanitation and Hygiene (WASH) Benefits Kenya study (clinicaltrials.gov identifier NCT01704105). Details of the parent trial (23) and maternal milk substudy (13) have been reported previously. In brief, the cluster-randomized parent trial was conducted in Kakamega, Bungoma and Vihiga counties in western Kenya from 2012 until 2016, and enrolled 8246 study families. The primary objectives of the parent trial were to assess the combined and individual effects of improved water, sanitation, hygiene, and nutrition on child growth and diarrhea in the first 2 y of life. Improved water, sanitation, and hygiene entailed provision of chlorine for water treatment, improved latrine facilities, and tools for safe management of children's feces, and 2 hand-washing stations/household, respectively. Improved nutrition included peer counseling on the WHO Infant and Young Child Feeding guidelines and provision of a lipid-based nutrient supplement to infants from 6 to 24 mo. All arms had a behavior change component to promote the target behaviors and each household received monthly visits from community-based promoters. Pregnant women in their second and third trimesters were eligible for enrollment in the parent trial. Study households invited to participate in the biochemical substudy (*n* = 1486) and the maternal milk substudy (*n* = 300) were evenly balanced across 4 intervention arms: *1*) Control; *2*) Water, Sanitation, and Hygiene; *3*) Nutrition; and *4*) Water, Sanitation, Hygiene, and Nutrition. Mothers between 1 and 6 mo postpartum were eligible for participation in the maternal milk substudy; hence, no children in the milk substudy were age eligible to receive the lipid-based nutrient supplement.

We received ethical approval from the Kenya Medical Research Institute Scientific Steering Committee and Ethical Review Committee, and the University of California, Berkeley Committee for the Protection of Human Subjects, upon which the University of California, Davis Institutional Review Board has relied for this research project. Study participants provided written consent for all research activities.

#### Data collection

At enrollment, study mothers reported their education level and marital status. At the time of milk collection, birth characteristics, including number of antenatal visits attended, parity, place of delivery of study child, infant birth weight, date of birth, and sex, were reported. Maternal age and anthropometry, along with maternal dietary data using a 7-d FFQ and an interactive 24-h recall, were also collected (13) at the time of milk sampling. Self-reported infant feeding practices, food security using the Household Hunger Scale (24), and season were also assessed during the milk collection visit, which took place 1–2 d after the infant serum collection date. The infant feeding questionnaire asked about liquids and solids that had been fed to the study child in the past 24 h and the past week.

Milk collection has been described previously (13). In brief, 5 mL of milk was collected via hand expression following 90 min observed nonbreastfeeding at 1 min into a feed from the right breast. Collection was done between 0900 and 1200 to minimize diurnal effects (25, 26), although recent findings suggest that milk vitamin B-12 concentration does not vary across time of day of collection (27). Milk specimens were stored on ice for ≤8 h, frozen at –4°C for ≤3 wk, and then transported to a –80°C freezer where they were stored until they were shipped on dry ice to the USDA, ARS, Western Human Nutrition Research Center in Davis, CA, for processing and analysis. Specimens were protected from sunlight: collection was restricted to dimly lit areas and cryovials were stored in opaque freezer boxes.

Trained phlebotomists collected infant blood samples. A maximum of 5 mL of venous blood was collected from study children. Children were ineligible if they met any 2 of the following criteria for dehydration: restless or irritable, sunken eyes, drinks eagerly, skin pinch goes back slowly, or listless or unable to perform normal activity. Upon collection, phlebotomists gently inverted the serum tube 8–10 times, wrapped it in aluminum foil for light protection, and placed it in a cooler box for at least 30 min prior to centrifuging. Vials were centrifuged at 1354 × *g* for 15 min and 3 serum aliquots were separated before being returned to the cooler box with ice packs in the field. At the end of each day (<8 h after collection), serum samples were frozen at –20°C for ≤3 wk, then transported to a –80°C freezer in Nairobi where they remained until they were shipped on dry ice to the Western Human Nutrition Research Center.

#### Milk and serum vitamin B-12 analysis

Milk samples were analyzed as described previously (28). The specimens were thawed at room temperature before centrifugation to separate the whey fraction. This whey fraction then underwent a heat treatment in the presence of dithiothreitol and potassium cyanide to disassociate haptocorrin from vitamin B-12. Finally, analysis by solid-phase competitive chemiluminescent enzyme immunoassay was done using a Siemens IMMULITE 1000 automated bioanalyzer. Serum samples were analyzed on a Cobas e411 (Roche Diagnostics) by competitive protein binding chemiluminescence immunoassay.

#### Variable definition

Serum vitamin B-12 concentrations were considered adequate (>220 pmol/L), depleted (148–220 pmol/L), or deficient (<148 pmol/L) based on established cutoff values for adults (2, 29) because infant cutoffs are not available. An animal source food score was constructed from a food frequency questionnaire that asked how many days out of the last 7 a mother consumed: *1*) poultry; *2*) flesh meat (cow, goat, and pig); *3*) organ meat; *4*) eggs; *5*) yogurt; *6*) tea with milk; *7*) milk alone; *8*) fresh fish; *9*) dried fish; and *10*) termites. The maximum score was 70 if a mother responded that each of the 10 items was consumed on all of the past 7 d. Scores were categorized into quartiles to reduce the skewness. The data collection (maternal milk, infant serum, and dietary information) was categorized by season; harvest or dry season was June–September, and the rainy season was October–November. All infant feeding designations are based on the prior 24 h. Exclusive breastfeeding was defined as only human milk being provided, except for oral rehydration salts, drops, or vitamin and mineral syrups (30). Predominant breastfeeding was defined as when maternal milk was the predominant source of nourishment and the infant also received other liquids, such as water-based drinks and fruit juices but not animal milks (30). Mixed feeding was defined as feeding maternal milk, other liquids (including nonhuman milks), and solids.

#### Statistical power and analysis plan

We calculated a sample size of 170 to be sufficient to detect a correlation of ≥0.25 between maternal milk vitamin B-12 (expressed as pmol/L) and infant serum vitamin B-12 concentration (expressed as pmol/L) with 90% power and a significance level of 0.05, accounting for a design effect of 1.35 (cluster size of 8 women, and intracluster correlation coefficient estimate = 0.05). A kernel density plot is used to display infant serum vitamin B-12 concentration, depicting the chance that the serum vitamin B-12 concentration falls within a particular range of values. Variables hypothesized to be associated with infant serum vitamin B-12 adequacy included child age, maternal milk vitamin B-12 concentration, season of data collection, antenatal care visit frequency as a proxy of wealth, maternal age, child sex and birth weight, maternal parity and education, household hunger, maternal recent animal source food intake and vitamin B-12 intake in the previous 24 h, and breastfeeding status. These characteristics were used to model adequate serum vitamin B-12 using logistic regression, with generalized estimating equations to account for correlated observations at the village cluster level (31). Covariates that were considered significant by *P* < 0.1 in bivariate logistic regression models were included in the multivariable regression model. Child age (continuous, in months) and intervention arm were forced into the multivariable model based on known biological of association with vitamin B-12 concentration (32) and to account for the study design, respectively. Data analysis was performed in SAS version 9.3 (SAS Institute Inc.) and figures were generated in R 3.3.1 software (R Core Team).

## Results

### 

#### Maternal and infant characteristics

Although complete data were available for 286 women participating in the maternal milk subsample, only 182 (64%) of them had corresponding infant serum samples available for laboratory analysis in this study. The lack of blood available for analysis was due to refusals, inability to draw blood, and insufficient volume collected. There were 39 (14%) mothers from the breast milk sample who did not consent to infant blood draw; no collection was possible for 19 (6%) infants; and 46 (16%) blood collections had insufficient volume, precluding serum analysis for vitamin B-12. Of the 182 serum samples available for analysis, 6 were excluded either because they were outliers (*n* = 4, 3 with concentration = 0 pmol/L and 1 with concentration >1000 pmol/L) or because infant feeding data were missing (*n* = 2), leaving 176 paired mother-child observations for the analytic sample of this study.

Participant characteristics were not significantly different between the larger maternal milk subsample and the dyads that had infant serum available for analyses (**Table 1**). Average maternal age was 26 y and that of infants was ∼4 mo. The majority of mothers attended 2–5 antenatal care visits during their pregnancy with the study child and most births (∼60%) took place at a health facility. Specimen (milk and serum) data collection was conducted predominantly during the harvest season and 70% of study households reported little or no household hunger in the month prior to interview. The median maternal vitamin B-12 intake in the 24 h prior to milk collection was 1.6 µg/d and 36% of women were consuming at or above the Recommended Daily Allowance of vitamin B-12 during lactation (2.6 µg/d). Exclusive breastfeeding was reported among less than a third of dyads and predominant breastfeeding was rare (10% or less). Mixed feeding was the most commonly reported infant feeding practice, and the most common food item other than mother's milk fed to infants was thin porridge (uji, data not shown).

**TABLE 1:** Characteristics of women and infants in the maternal milk subsample and in the analytic sample for infant serum analysis within the WASH Benefits Study, Kenya^1^

	Maternal milk	Infant serum analytic
Characteristics	subsample (*n* = 286)	sample (*n* = 176)
Child age, mo	3.7 ± 1.3	3.9 ± 1.3
Maternal age, y	26.1 ± 5.5	26.5 ± 6.1
Child sex is male	127 (44)	77 (45)
Child birthweight,^2^ kg	3.3 ± 0.6	3.3 ± 0.7
Maternal BMI, kg/m^2^	22.7 ± 3.1	22.5 ± 2.9
Maternal parity	3.5 ± 2.0	3.6 ± 2.0
Number of antenatal visits	3.6 ± 1.1	3.6 ± 1.2
Place of delivery		
Home	105 (37)	70 (40)
Health facility	181 (63)	106 (60)
Marital status		
Married, cohabitating	260 (91)	160 (91)
Married, living apart	24 (8)	15 (9)
Not married	2 (1)	1 (<1)
Maternal education		
None or primary	228 (80)	142 (81)
Secondary or beyond	58 (20)	34 (19)
Household hunger		
Little or none	208 (73)	123 (70)
Moderate or severe	78 (27)	53 (30)
Season^3^		
Dry (harvest)	220 (77)	138 (78)
Rainy	66 (23)	38 (22)
Maternal animal source food score, item-d/wk		
Quartile 1 (0–6)	62 (22)	41 (23)
Quartile 2 (7–9)	79 (28)	48 (27)
Quartile 3 (10–13)	75 (26)	46 (26)
Quartile 4 (14–30)	70 (24)	41 (23)
Maternal vitamin B-12 intake, µg/d	1.5 [0.3, 9.7]	1.6 [0.3, 10.3]
Breastfeeding status (last 24 h)		
Exclusive breastfeeding	87 (30)	47 (27)
Predominant breastfeeding	23 (8)	18 (10)
Mixed feeding	176 (62)	111 (63)

^1^Values are mean ± SD, median [IQR], or *n* (%). WASH, Water Sanitation and Hygiene.

^2^Birthweight data limited in maternal milk subsample (*n* = 109) and serum subsample (*n* = 73).

^3^Season of biospecimen (milk, serum) collection. Dry (June–September); rainy (October–November).

#### Infant serum vitamin B-12 concentration

**FIGURE 1:**
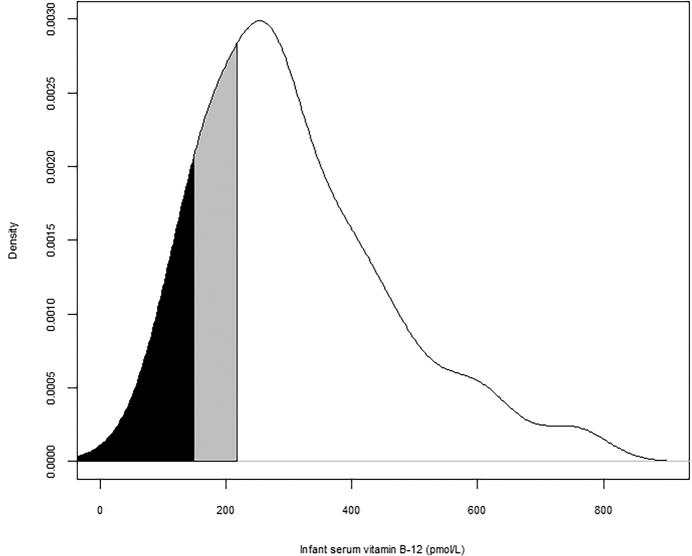
Kernel density plot of infant serum vitamin B-12 concentration within a subsample of the WASH Benefits, Kenya biochemical substudy (*n* = 176). The areas shaded black and gray represent the proportion of the population that had serum vitamin B-12 concentration <148 pmol/L (indicative of deficiency), and >148 pmol/L but <220 pmol/L (indicative of depletion), respectively. The *y*-axis values, density, represent the relative likelihood of vitamin B-12 concentration among this population (e.g., a density of 0.002 multiplied by 150 pmol/L = 0.3); the AUC sums to 1. WASH, Water Sanitation and Hygiene.

The median infant serum vitamin B-12 concentration was 279 pmol/L, IQR 195–401 pmol/L (**Figure 1**). Approximately 10% of infants had a serum vitamin B-12 concentration indicating deficiency (<148 pmol/L), 20% had marginal concentrations, indicating vitamin B-12 depletion (148–220 pmol/L), and 70% had concentrations considered adequate (>220 pmol/L).

#### Correlation between infant serum and maternal milk vitamin B-12

**FIGURE 2:**
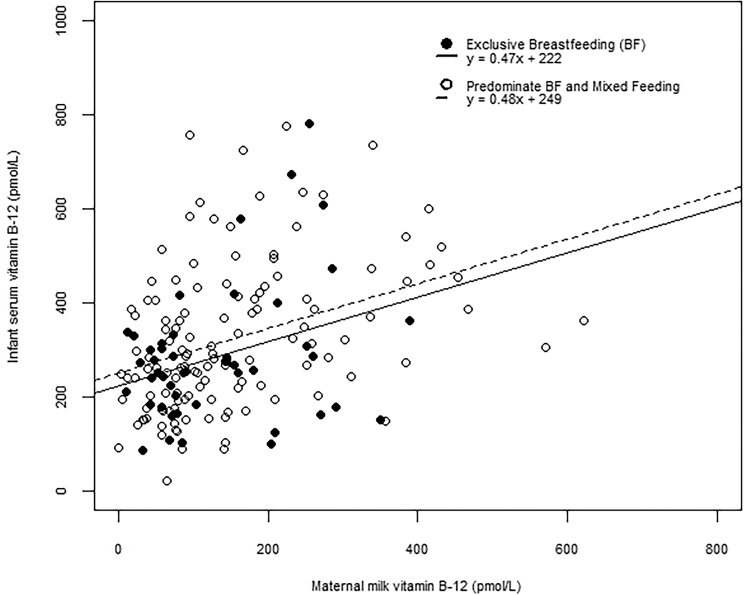
Relation between vitamin B-12 in infant serum and maternal milk by reported infant feeding practice among a subsample of the WASH Benefits, Kenya biochemical substudy. The correlation for the relation between infant serum vitamin B-12 and maternal milk vitamin B-12 (both expressed in pmol/L) was similar for exclusive breastfeeding (*r* = 0.32, *n* = 47) and the combined predominantly breastfeeding and mixed feeding groups (*r* = 0.37, *n* = 129). WASH, Water Sanitation and Hygiene.

We detected a positive correlation between maternal milk vitamin B-12 and infant serum vitamin B-12 (*r* = 0.36, *P* < 0.001). We also performed a subgroup analysis by separating the mother-infant dyads by self-reported exclusive breastfeeding. The correlation coefficients were significant and similar irrespective of exclusive breastfeeding behavior. There was a 0.47 pmol/L (95% CI: 0.05, 0.90 pmol/L) increase in infant serum vitamin B-12 per 1 pmol/L increase in maternal milk vitamin B-12 among exclusively breastfed infants and a corresponding 0.48 pmol/L (95% CI: 0.26, 0.69 pmol/L) increase among mixed-fed or predominately breastfed infants (**Figure 2**).

In previous analyses, we found that ∼10% of these mothers had milk vitamin B-12 concentrations >310 pmol/L, which is calculated from the AI for infants that are exclusively breastfed (13). Of the 20 mother-infant pairs with concentrations >310 pmol/L, 80% of infants had serum vitamin B-12 concentrations considered adequate, 15% of infants had serum vitamin B-12 concentrations considered marginal, and 1 infant had a serum vitamin B-12 concentration indicating deficiency.

#### Characteristics associated with adequate infant serum vitamin B-12

In bivariate analyses, higher quartiles of maternal milk vitamin B-12 concentration predicted greater odds of infant vitamin B-12 adequacy (*P*-trend < 0.01). Sample collection performed during the rainy season was associated with lower odds of vitamin B-12 adequacy (>220 pmol/L) in infants. The frequency of antenatal visits during pregnancy was associated with greater odds of serum vitamin B-12 adequacy (>220 pmol/L) among infants. There was no evidence to suggest that child age, birthweight, household hunger, or the maternal dietary patterns that we measured were associated with serum vitamin B-12 adequacy in infants (**Table 2**). In adjusted analyses, maternal milk vitamin B-12 concentration was the only factor that remained statistically significant (*P*-trend = 0.03). For mothers in the highest quartile of milk vitamin B-12 concentration (≥210 pmol/L), there was a 270% greater odds of adequate infant vitamin B-12 status (AOR 2.7; 95% CI: 0.9, 7.4).

**TABLE 2:** Characteristics associated with adequate serum vitamin B-12 (>220 pmol/L) among infants 1–6 mo old in a subsample of the WASH Benefits Study, Kenya^1^

	Unadjusted	Adjusted
Characteristics	OR (95% CI)	OR (95% CI)^2^
Child age, mo	1.2 (0.9, 1.6)	1.2 (0.9, 1.5)
Maternal milk vitamin B-12		
Quartile 1 (0–64 pmol/L)	REF	REF
Quartile 2 (65–110 pmol/L)	1.1 (0.5, 2.4)	0.9 (0.4, 2.1)
Quartile 3 (111–209 pmol/L)	2.4 (0.8, 7.6)	2.1 (0.6, 6.6)
Quartile 4 (210–862 pmol/L)	3.3 (1.2, 8.4)	2.7 (0.9, 7.4)
Sampled in rainy season^3^	0.4 (0.2, 0.9)	0.7 (0.3, 1.5)
Number of antenatal visits	1.3 (1.1, 1.8)	1.2 (0.9, 1.7)
Intervention arm		
Control	REF	REF
Water, Sanitation, Hygiene	0.5 (0.2, 1.2)	0.4 (0.2, 1.1)
Nutrition	0.2 (0.1, 0.7)	0.5 (0.2, 1.3)
Water, Sanitation, Hygiene, Nutrition	0.7 (0.3, 1.9)	0.8 (0.3, 2.4)
Maternal age, y	1.0 (0.9, 1.0)	—
Child sex is male	1.1 (0.5, 2.2)	—
Child birthweight,^4^ kg	1.5 (0.7, 3.2)	—
Primiparous	1.8 (0.8, 4.2)	—
Place of delivery		
Health facility	REF	—
Home	1.2 (0.6, 2.2)	—
Maternal education		
None or primary	REF	—
Secondary or beyond	0.8 (0.3, 1.7)	—
Household hunger		
Little or none	REF	—
Moderate or severe	1.5 (0.8, 3.0)	—
Maternal animal source food score, item-d/wk		
Quartile 1 (0–6)	REF	—
Quartile 2 (7–9)	1.1 (0.4, 2.5)	—
Quartile 3 (10–13)	0.6 (0.2, 1.3)	—
Quartile 4 (14–30)	0.5 (0.2, 1.3)	—
Maternal vitamin B-12 intake		
<1.6 µg/d	REF	—
≥1.6 µg/d	0.4 (0.2, 1.1)	—
Breastfeeding status (last 24 h)		
Exclusive breastfeeding	REF	—
Predominant breastfeeding or mixed feeding	0.7 (0.3, 1.4)	—

^1^Characteristics were modeled with binary regression using generalized estimating equations to account for correlation at the cluster level. *n* = 176. WASH, Water Sanitation and Hygiene.

^2^Adjusted model includes characteristics known to be related to vitamin B-12 adequacy (child age), intervention arm and variables with *P* < 0.1 in bivariate analyses (antenatal visit frequency, season, maternal milk vitamin B-12 quartile).

^3^Dry season (June–September); rainy season (October–November).

^4^Child birthweight (*n* = 73).

## Discussion

In this study of 1- to 6-mo-old infants in rural western Kenya, we found a positive correlation between maternal milk and infant serum vitamin B-12 concentration that did not differ by reported exclusive breastfeeding in the last 24 h. We found no biological, demographic, or dietary characteristics associated with infant serum vitamin B-12 adequacy aside from maternal milk vitamin B-12 concentration. However, maternal vitamin B-12 status was not assessed and we presume that it likely contributed to both infant vitamin B-12 stores in utero as well as milk vitamin B-12 concentration (33, 34).

To our knowledge, there is only one previous report of the positive correlation between maternal milk and infant serum vitamin B-12 concentrations similar to what was found in our study (*r* = 0.36, *P* < 0.001). Greibe et al. (32) found a greater correlation between maternal milk and infant serum (*r* = 0.58) with a smaller sample (*n* = 25) of exclusively breastfed Danish infants at 4 mo of age. Our study is unique because we had a larger sample of unsupplemented mother-infant dyads (*n* = 176) and there was a variety of reported infant feeding practices, even though all infants were in the age range when exclusive breastfeeding is recommended (20). We restricted the sample to those reporting exclusive breastfeeding in order to determine if there was a stronger association among that subset of mother-infant dyads, but did not find that the relation changed notably. The study was powered to detect a correlation of ≥0.25 and our Pearson's correlation coefficient was >0.25 for the full sample and the exclusively breastfeeding mother-infant dyads. Although the correlation is statistically significant between these 2 biomarkers, it is not strong, which suggests that there are other drivers of infant vitamin B-12 status.

One contributor to vitamin B-12 status is the capacity to adequately absorb vitamin B-12, and among older adults malabsorption of vitamin B-12 is a common cause of deficiency (4, 29). Among infants with a maturing gut and greater susceptibility to infection, environmental enteropathy is a plausible mechanism for contributing to poor vitamin B-12 absorption. A bacterial infection with *Heliocampter pylori*, and resultant lower acidity of the stomach, has been shown to decrease absorption of vitamin B-12 (35). However, among our population of infants, we saw higher than expected vitamin B-12 concentrations when examining breast milk, suggesting that either receipt of cow milk among mixed-fed infants or prior stores from birth are contributing to status. There is mounting evidence to suggest that maternal vitamin B-12 status, especially during pregnancy, may be a better predictor of infant vitamin B-12 status than maternal milk vitamin B-12 concentration (8, 33, 34). Unfortunately, we did not measure vitamin B-12 in maternal serum.

There was insufficient evidence to draw many conclusions from the analysis of covariates related to serum vitamin B-12 adequacy other than the positive relation between infant serum and maternal milk vitamin B-12 concentration. Other studies have shown plasma vitamin B-12 concentrations to be positively correlated with socioeconomic status (11, 12) but the proxy variables used to determine socioeconomic status in our study were not correlated with infant vitamin B-12 adequacy. We did find that older children tended to have increased odds of vitamin B-12 adequacy (*P* = 0.06).

Among infants, there was a relatively low prevalence of vitamin B-12 deficiency or depletion (30%). This finding was surprising given that most (∼90%) mothers had low milk vitamin B-12 concentrations (<310 pmol/L) (13). It is notable that our results are similar to those reported by Chebaya et al. (36), who also found infant vitamin B-12 deficiency to be uncommon, although there was a high prevalence of mothers with milk vitamin B-12 concentrations <362 pmol/L. Among exclusively breastfed infants in Canada and Cambodia, 6–16% had vitamin B-12 <220 pmol/L, while one-half and three-quarters of Canadian and Cambodian mothers, respectively, had milk vitamin B-12 <362 pmol/L (36). We speculate that infant consumption of cow milk and potential bias, or overreporting of exclusive breastfeeding practices, could explain the discrepancy between infant status and maternal breast milk vitamin B-12 concentrations.

The relatively low prevalence of infant vitamin B-12 deficiency compared with the majority of mothers having milk vitamin B-12 concentrations <310 pmol/L raises a number of questions. First is the question of infant diet. Many of these infants (73%) were not exclusively breastfed, so the contribution of vitamin B-12 from foods other than maternal milk could be an explanation for the apparent lack of infant vitamin B-12 deficiency. Indeed, of the children classified as mixed fed, approximately one-third were fed a milk product the day prior to specimen collection. And although we did not find a significant difference in vitamin B-12 adequacy by reported infant feeding status, others have reported that mixed feeding during infancy correlated to a better vitamin B-12 status than infants solely fed breast milk (32). In our study, milk products and wheat products were the second most common food items after maize-based porridge, reportedly fed to 12% of mixed-fed infants on the 24-h infant feeding food frequency questionnaire. At this point of data collection, quantitative multipass infant 24-h dietary recall was not done to capture quantity of foods consumed. Therefore, we cannot estimate the contribution of cow milk to infant vitamin B-12 status quantitatively. It is also important to note that exclusive breastfeeding may have been overreported on the basis of social desirability bias, which has been demonstrated (37). A component of the behavioral intervention in the nutrition arms of this trial and standard of care counseling emphasizes the importance of exclusive breastfeeding during the first 6 mo of infant life. The second question is on the certainty of our vitamin B-12 cut-points for infants. Unfortunately, the cut-points for classifying infant vitamin B-12 status and maternal milk vitamin B-12 adequacy may not be valid. The cut-points used for infant depletion, deficiency, and adequacy were derived for adults (2) and the milk adequacy concentration is based on AI estimates from a small sample of women and agreement with a single small study assessing urinary methylmalonic acid as a functional outcome (13, 38). Hay et al. (17) published a reference interval ranging from the 5th to 95th percentiles for serum vitamin B-12 concentrations of 6-mo-old Norwegian breastfed infants that spanned 121–517 pmol/L. While these children were not exclusively breastfed and therefore may be a good comparison for the predominantly mixed-fed Kenyan infants in this study, we would expect that these children may have been exposed to formula that is fortified with vitamin B-12. Overall, more work is needed that includes infant functional outcomes in order to better classify vitamin B-12 status in lactation and infancy.

Our analyses are limited because we did not measure maternal serum vitamin B-12 during pregnancy or lactation and the data are cross-sectional. To define exclusive breastfeeding via the last 24 h of feeding does not accurately describe longitudinal intake, as women often go in and out of exclusive breastfeeding (39). Therefore, infants defined as exclusively breastfed may have received animal-source foods, such as milk, prior to the past 24 h. However, the current status measurement that captures behaviors of breastfeeding in that past 24 h is the most common for surveys (39). Another limitation of our study is that we did not measure a wider panel of vitamin B-12 biomarkers in infants, such as homocysteine, methlymalonic acid, or transcobalamin. Two strengths of this study include that the laboratory methods were performed using the most up-to-date methods for analysis (28), and the population sampled has dietary patterns consistent with much of sub-Saharan Africa (low dietary diversity and a high proportion of energy intake from staple crops). Hence, our findings may be generalizable to areas where there is low animal-source food intake among women during pregnancy and lactation, infants are introduced to nonhuman milks before 6 mo old, and maize-based porridge is the predominant infant food (40, 41). Neumann et al. (14) found similarly low maternal milk vitamin B-12 concentrations in Kenya from samples collected over 2 decades ago, but their results must be interpreted with caution because the laboratory methods to assess vitamin B-12 did not account for interferences due to haptocorrin.

There is a risk of vitamin B-12 deficiency for infants among populations consuming limited animal-source foods, but we have limited tools to assess infant deficiency. The contribution of human milk to infant vitamin B-12 status is important to quantify, given that maternal milk vitamin B-12 concentration increases with maternal supplementation (15). Yet, the contribution of maternal vitamin B-12 to infant status may depend more on maternal stores during pregnancy than on her intake or status during lactation, which elevates the importance of maternal nutrition across the stages of pregnancy and lactation.
